# Sella Turcica Morphology on Cephalometric Radiographs and Dental Abnormalities—Is There Any Association?—Systematic Review

**DOI:** 10.3390/ijerph18094456

**Published:** 2021-04-22

**Authors:** Tomasz Jankowski, Maciej Jedliński, Katarzyna Grocholewicz, Joanna Janiszewska-Olszowska

**Affiliations:** 1 Private Practice Dental Clinic Jankowscy, 68-200 Żary, Poland; tomaszjankowski1905@gmail.com; 2 Department of Interdisciplinary Dentistry, Pomeranian Medical University in Szczecin, 70-204 Szczecin, Poland; maciej.jedlinski@pum.edu.pl (M.J.); katgro@pum.edu.pl (K.G.)

**Keywords:** sella turcica, dental abnormalities, sella bridging

## Abstract

Background: The sella turcica is a saddle-like structure in the middle cranial fossa on the intracranial surface of the sphenoid bone, visible on lateral cephalograms routinely conducted for orthodontic diagnosis. The development of facial structures follows similar traits to the sella turcica: glandular anomalies may be associated with functional disorders, e.g., altered hormonal levels, thus influencing dental development. The aim of this study is to find out if there is any association between the morphology of the sella turcica on cephalometric radiographs and the presence of dental abnormalities. (2) Methods: The search was conducted on 27 January 2021 in four search engines: Medline (PubMed Central), Scopus, Web of Science, Embase. The keywords used in the search strategy were as follows: “sella turcica” AND (“dental abnormalities” OR “dental anomalies” OR “malocclusion”). Since all the studies finally included were retrospective case–control studies, the Newcastle–Ottawa Quality Assessment Form for Case–Control Studies was applied. (3) Results: The search strategy identified 465 articles: 289 from PubMed, 121 from Scopus, 32 from Web of Science and 23 from Embase. Finally, 10 full-text papers were included into qualitative analysis. (4) Conclusions: Sella turcica bridging is very frequent among orthodontic patients. A clear association exists between dental abnormalities and sella turcica bridging.

## 1. Introduction

The sella turcica is a saddle-like anatomical structure in the middle cranial fossa on the intracranial surface of the sphenoid bone, visible on lateral cephalograms routinely conducted for orthodontic diagnosis [1]. Its anterior border is the tuberculum sellae and the posterior border is the dorsum sellae [2]. The pituitary gland is located in the pituitary fossa and consists of the anterior lobe (adenohypophysis), intermediate lobe and posterior lobe (neurohypophysis) [3].

The prenatal and postnatal developments of the sella turcica and pituitary gland are interrelated since the formation of the pituitary gland must be completed before the sella turcica can be created. Thus, anomalies of the gland modify the sellar morphology [4]. The anterior part develops mainly from neural crest cells that are not directly related to the notochord. The posterior part develops from the paraxial mesoderm, which is dependent on the notochord [5]. Abnormalities in the anterior sellar wall might be linked to anomalies in the frontonasal fields, whereas those in the posterior wall seem to be related to brain malformations [4].

The development of facial structures follows similar traits to the sella turcica: with a high importance of neural crest cells and mesodermal cells [6]. The development of the midface, including the sella turcica and teeth, may be modified by disrupting signaling pathways due to mutations in the homeobox genes [7]. Moreover, glandular anomalies may be associated with functional disorders, e.g., altered hormonal levels, thus influencing dental development [8].

Dental abnormalities may refer to the dental morphology, dental development, position of eruption or number of teeth. The most prevalent dental abnormality is hypodontia with occurrence ranging from 1.6% to 36.5%, depending on the population studied [9]. Various terms have been used to describe hypodontia: “congenitally missing teeth”, “tooth agenesis”, “oligodontia” and “anodontia” [9]. Hypodontia is more common in females [10,11]. Badrov et al. reported that dental development was more delayed in children with congenitally missing permanent teeth than in the control group [12]. Hyperdontia is diagnosed if supernumerary teeth are present. Its prevalence in Caucasians ranges between 0.15% and 3.9% [13]. “Concomitant hypo-hyperdontia” is a rare numerical mixed discrepancy: some teeth may be supernumerary, and some others may be absent [13].

Tooth transposition, e.g., an interchange in the position of two permanent adjacent teeth located at the same quadrant in the dental arch, is a unique and severe condition of ectopic eruption with incidence in the overall population from 0.2% to 0.38% [14].

Dental agenesis may be associated with impaired masticatory function as well as alveolar bone deficiency. When primary teeth are congenitally missing, development of the permanent dentition is often delayed [12,15,16,17]. A delayed dental development can negatively influence patients’ self-esteem and interfere with orthodontic treatment plans [12,15,16,17].

The aim of this study is to find out if there is any association between the morphology of the sella turcica on cephalometric radiographs and the presence of dental abnormalities.

## 2. Materials and Methods

### 2.1. Literature Search

**Figure 1:**
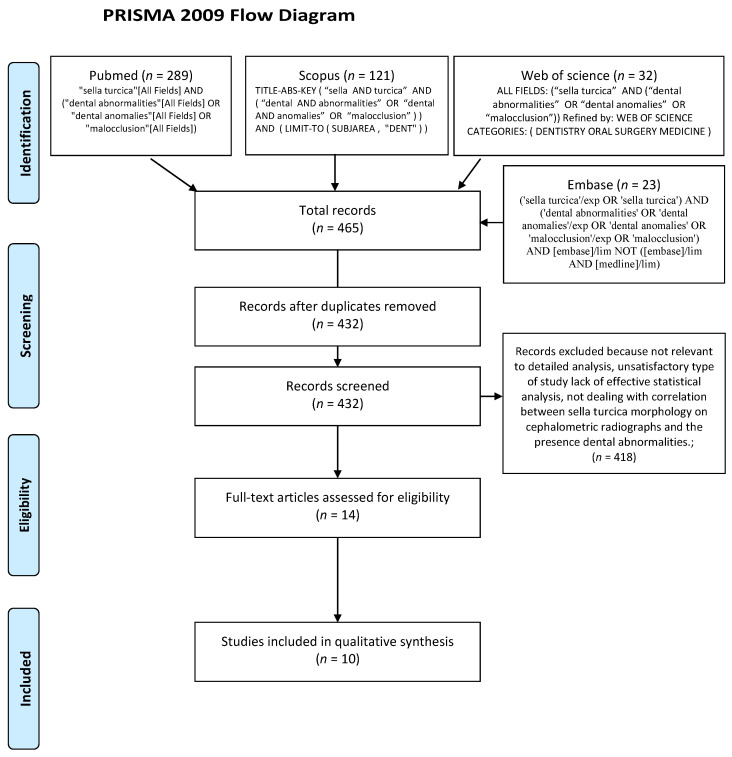
Prisma 2009 flow diagram.

The search was conducted on 27 January 2021 in 4 popular search engines: Medline (PubMed Central), Scopus, Web of Science, Embase. All searching was performed using a combination of subject headings, MeSH terms and free-text terms. The final search strategy was established through several pre-searches. The keywords used in the search strategy were as follows: “sella turcica” AND (“dental abnormalities” OR “dental anomalies” OR “malocclusion”). The search strategy for MedLine (PubMed Central), Scopus, Web of Science and Embase is presented in Figure 1 (Prisma 2009 flow diagram). Reference lists of primary research reports were cross-checked in an attempt to identify additional studies.

### 2.2. Eligibility Criteria

Studies were included in the review if they referred to the correlation between the sella turcica morphology on cephalometric radiographs and the presence of dental abnormalities. In order to ensure the best quality of evidence, only randomized clinical trials, case–control studies and cohort studies were included. There were no time limits for the year of publication introduced.

The exclusion criteria were as follows:

1. Lack of effective statistical analysis;
2. Reviews;
3. Case reports and case series;
4. Abstract and author debates or editorials;
5. Studies written in a language other than English.

### 2.3. Data Extraction

Titles and abstracts found during the search were studied and selected for further analysis independently by two authors (M.J. and T.J.). The full text of each identified primary included article was then analyzed to prove whether it fitted the eligibility criteria. Disagreements were resolved through discussion with the team supervisor (J.J.O.). Authorship, year of publication, data concerning methods, type of dental anomalies, reference landmarks used in cephalometry and measurements taken were independently extracted by two authors (T.J. and M.J.) and examined by the supervisor (J.J.O).

### 2.4. Risk of Bias

According to the PRISMA statements, the evaluation of methodological quality provides an indication of the strength of evidence provided by the study because methodological flaws can result in bias [18]. Due to the fact that all the studies that were finally included in the review were retrospective case–control studies, finally, only the Newcastle–Ottawa Quality Assessment Form for Case–Control Studies was applied. In the Newcastle–Ottawa Quality Assessment Form, the quality of all included case–control studies was based on object selection, comparability and exposure. The possible quality assessment score ranged from zero to nine points, with a high score indicating a good-quality study. For selection, the maximum number of points, if all criteria were met, was four, for comparability, it was two and for outcome, it was three [19,20].

## 3. Results

### 3.1. Search Results

The search strategy identified 465 potential articles: 289 from PubMed, 121 from Scopus, 32 from Web of Science and 23 from Embase. After duplicates had been removed, 432 articles were screened. Then, 418 papers were excluded because they did not correspond to the topic of this review. Of the remaining 14 papers, 4 were excluded because they were not relevant to the eligibility criteria. Finally, 10 full-text papers were included into qualitative analysis (Figure 1 PRISMA 2009 flow diagram). Data concerning the frequency of sella turcica morphological types in the general population and in individuals with dental anomalies were extracted and tabularized. The characteristics of each included study are presented in Table 1. 

All the studies included refer to sella turcica bridging. It is evident that the prevalence of this sella type (considered as an abnormality) is very high in the groups of patients without dental abnormalities (control groups) of the cephalometric studies included, ranging from 25% [25] to 57% [26]. Moreover, it should be noticed that, even in the CBCT study included [22], sella turcica bridging occurred in 50% of the control group.

Concerning dental abnormalities, the authors of the studies included analyzed the following: palatally displaced canines [21,22,27], impaction or transposition of canines or premolars [23], congenitally missing lateral incisors [24], impacted canines and hyperdontia [25], impacted canines [26], dental agenesis [26,29], hyperdontia [26], maxillary or mandibular dental transposition [28], congenitally missing second mandibular premolars or the presence of palatally displaced canines [30]. Thus, in most studies, the study groups were non-homogenous. Almost all the studies included showed significant differences between the study (with dental abnormalities) and control groups.

Leonardi et al. [30] compared a sample of subjects with dental abnormalities (PDC: palatally displaced canine, or congenital absence of the mandibular second premolar) to a group of individuals without dental abnormalities. A complete sella turcica bridge was found in 17.6% of adolescents with dental abnormalities and in 9.9% of adolescents without dental abnormalities.

In another study, Leonardi et al. [28] stated that a sella turcica bridge is more frequent in subjects with dental transposition than in the control group. Similar results have been reported by other authors who investigated correlations between sella turcica bridging and dental abnormalities using lateral and panoramic radiographs [21,23,24,25,26,27,29].

A contrary conclusion has been drawn in a CBCT study by Ortiz et al. [22], who confirmed no statistically significant correlation between palatal canine impaction and sella turcica bridging.

### 3.2. Risk of Bias

The results of the quality assessment are presented in Table 2.

### 3.3. Effect Size

**Figure 2:**
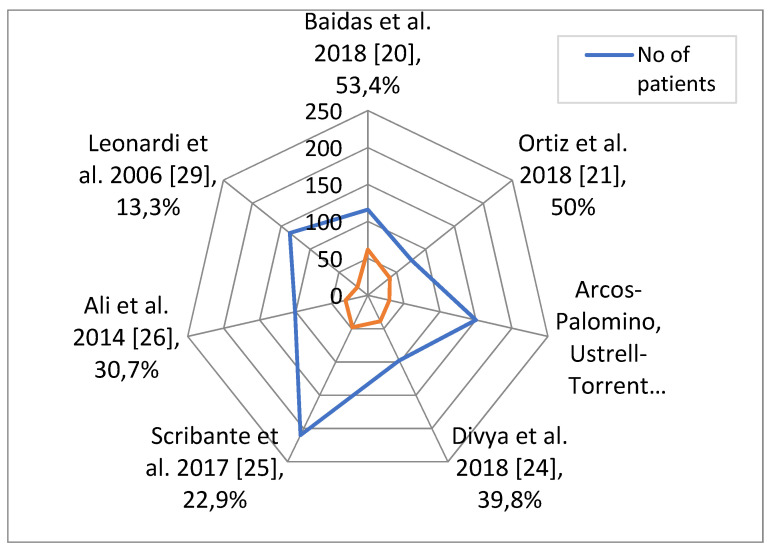
Total number of patients included in each study, and numbers and percentages of patients with PDC.

**Figure 3:**
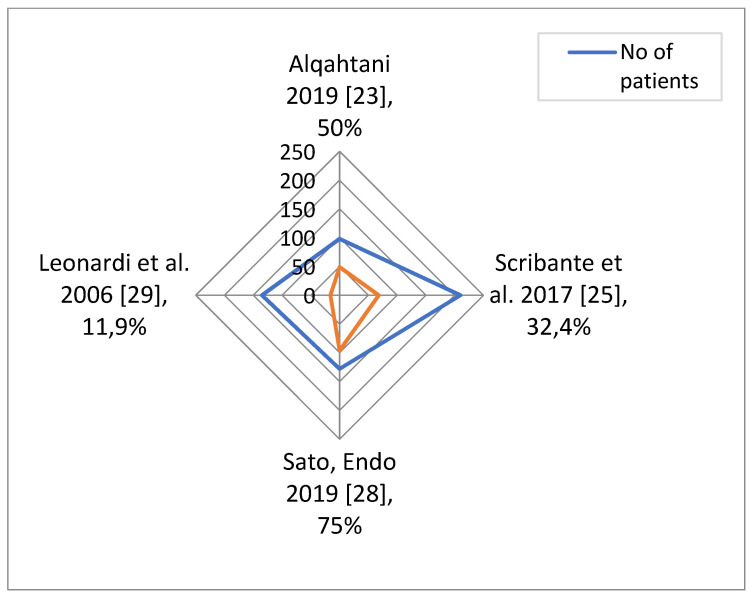
Total number of patients included in each study, and numbers and percentages of patients with hypodontia.

In order to establish whether dental abnormalities occur more frequently in populations of patients with sella turcica bridging, seven studies for PDC and four studies for hypodontia were taken into account. The total number of patients included and numbers and percentages of patients with dental abnormalities are presented in Figure 2 and Figure 3. The data concerning the prevalence of dental abnormalities in the general population were extracted from an epidemiological study on 4702 healthy individuals [31]. A difference was considered significant at *p* < 0.05. The R statistical program (The R Foundation for Statistical Computing, Wirtschaftsuniversität Wien, Vienna, Austria) was used to perform calculations.

Means and standard deviations of the percentage of each abnormality weighted by the number of patients in each study were calculated. The weighted *t*-test was used to check if the difference in the abnormality percentage and the percentage typical for the general population (7.5% for PDC and 7.1% for hypodontia in the study on 4702 healthy individuals) is significant [31]. Results are shown in Table 3.

Hypodontia in the studied groups has a larger mean and larger between-studies standard deviations than PDC, which indicates its higher prevalence in patients with sella turcica bridging. The prevalence of both abnormalities in patients with sella turcica bridging is significantly higher than in the general population.

## 4. Discussion

It should be noted that the overall quality of the studies included in this review is high. The main problem that the researchers faced unsuccessfully was the selection of the study groups. Many of them used occasional selection of cases, without setting clear criteria for including a given case into the study. This calls into question the representativeness of the study groups and eventual clinical application of the conclusions of the study to the entire population. Frequent errors were as follows: lack of an error study in terms of inter- and intraexaminer reliability, small number of patients included into the study without justifying this with a sample size adjustment or lack of blinding of the person who performed the cephalometric analysis.

Possible limitations of this systematic review result from a lack of standards for keywords in scientific papers. The use of a combination of subject headings, MeSH terms and free-text terms: “sella turcica” AND (“dental abnormalities” OR “dental anomalies” OR “malocclusion”), makes it impossible to find scientific papers with very detailed multi-word keywords, such as Dadgar et al. 2020 [32] (“Palatally displaced impacted maxillary canines”, “Skeletal anomalies and normal variants”, “Sella turcica bridging”, “Atlas ponticulus posticus (arcuate foramen; sagittal foramen))” or Wak et al. 2018 [33], with the following keywords: “Sella turcica bridging”, “Palatal impacted canine”, “CBCT”. In our opinion, the use of multi-word keywords is a serious obstacle in finding scientific papers to be cited and thus should be discouraged by editors in authors’ instructions.

Axelsson et al. [34] described a normal sella turcica and five types of dysmorphology: oblique anterior wall, sella turcica bridging, double contour of the floor, irregularity (notching) in the posterior part of the dorsum sellae and pyramidal shape of the dorsum sellae. Kucia et al. [35] expanded the types of sella dysmorphology of three other variants: hypertrophic posterior clinoid process, hypotrophic posterior clinoid process and oblique contour of the floor.

Bridging is a fusion of the anterior and posterior clinoid processes [36]. Becktor et al. [37] classified sella turcica bridges into two variants: type A—manifest, ribbon-like fusion; type B—extension of the anterior and/or the posterior clinoid process (thin fusion anteriorly, posteriorly or in the middle). Another classification uses the degree of calcification of the interclinoid ligament (ICL): Class I (no calcification)—the sella turcica was longer than or equal to three fourths of its diameter; Class II (ICL partially calcified—incomplete bridge)—less than or equal to three quarters; Class III (ICL completely calcified—bridging)—radiographically visible diaphragma sella [30]. The diaphragma sellae is a straight line corresponding to the distance from the tuberculum sellae to the tip of the dorsum sellae.

The frequency of a complete sella turcica bridge in the literature is presented in Table 4 and Table 5. In studies on healthy individuals, a complete sella turcica bridge appears from 1.46% to 11.67%; a higher occurrence has been found in patients with dental abnormalities (6.45–33.30%).

A sella turcica bridge visible on lateral radiographs can signify a true bony union of the anterior and posterior processes or overlapping, which is difficult to determine [34]. Currently, 3D radiographs seem to be the most reliable study material [34]; however, according to the ALARA rule, CBCT cannot be a routine diagnostic tool in dentistry.

Moreover, Arcos-Palomino and Ustrell-Torrent [23] stated that sella turcica bridging was unrelated to sex, but it was significantly influenced by age. Thus, the prevalence of complete sellar bridging is lower in studies performed on adolescent patients [28,29]. Similarly, Caderberg et al. [42] confirmed that the degree of calcification of the sellar interclinoid and petroclinoid ligaments is age-dependent.

Sella turcica bridging was analyzed in all the studies included, since it appeared very frequently. It is very interesting that a high prevalence of sella turcica bridges was found in all the control groups of the studies included. The reason for such findings may be the inclusion of patients with different skeletal classes. A possible association between anomalies of the sella turcica and malocclusion has been reported by Kucia et al. [35], who proved that children with sella turcica abnormalities (mainly bridge) have more protruded incisors and a more distal position of the maxilla and mandible than a control group of patients with normal sella turcica morphology. Similarly, Motwani et al. [47] confirmed an association between sellar morphology and the type of malocclusion.

The fact that most of the studies included analyzed study groups consisting of patients with different dental abnormalities justifies the search for papers referring to “dental abnormalities”.

It seems strange that the only study not to find a statistically significant difference in the occurrence of sella turcica bridging between patients with and without palatally displaced canines is a study based on CBCT [23]. Similar findings have been reported by Wak et al. [33]. It is clear that cephalometric radiographs are taken routinely in most orthodontic patients. CBCT is typically used to assess the position of impacted teeth. A question arises referring to the control group, since no radiation may be used without clinical indications. In the study by Ortiz et al. [23], the control group consisted of patients with impacted third molars that required CBCT for clinical indications (previous to extractions), and no indication for CBCT in the control group is provided in the study by Wak et al. [33]. It may thus be supposed that control groups could be characterized by the presence of a pathology associated with a higher prevalence of sella turcica bridging than subjects with normal dentition and occlusion (with no clinical indications for CBCT). Another interesting finding is a significantly lower prevalence of sella turcica bridging found in the same patients, when comparing lateral cephalometric radiographs and CBCT, resulting from overlapping anatomical structures and sensitivity to alterations in head positioning, especially rotation [40].

Finally, it can be supposed that cranial morphology can also be influenced by other acquired anomalies in the course of various diseases [48,49]. Future studies concerning stem cells may improve the existing knowledge on the etiology of both dental and cranial alterations [50]. It should also be noted that the use of biomaterials can be a potential bias in evaluating dental anomalies [51,52].

## 5. Conclusions

(1) Sella turcica bridging on cephalometric radiographs is very frequent among orthodontic patients;
(2) A clear association exists between dental abnormalities (palatally displaced canines and hypodontia) and sella turcica bridging visible on cephalometric radiographs.

## Figures and Tables

**Table 1:** Characteristics of included studies.

Author and Year of Publication	Type of Study	Study Objective	Number of Subjects	Age Range (Years)	Type of Sella Turcica Abnormality	Type of Dental Abnormality	Verification	Results
Baidas et al. 2018 [21]	Case–control study	To test the association between sella turcica bridging with canine impaction using panoramic and cephalometric radiographs.	62 orthodontic patients with palatally impacted canines (study group) and 54 with erupted canines (control group).	12–25	Sella turcica bridging	Palatally impacted canines	15 lateral cephalograms were chosen at random and traced, and then retraced after interval of 3 weeks under identical conditions.	The frequency of sella turcica bridging is higher in subjects with palatally impacted canines (the occurrence of partial and complete bridging: study group 67.8%, control group 26%).
Ortiz et al. 2018 [22]	Case–control study	To investigate the association between unilateral/bilateral maxillary canine impaction and sella turcica bridging using CBCT.	38 subjects diagnosed with unilateral or bilateral palatal canine impaction (study group) and 38 without dental abnormalities (control group).	10–30	Sella turcica bridging	Unilateral or bilateral palatal canine impaction	One investigator remeasured 21 randomly selected scans from the impacted canine and control groups after a period of 4 weeks.	Sella turcica bridging occurred in 59.3% and 50% in the impacted canine and control groups, respectively. Thus, no statistically significant correlation has been confirmed between palatal canine impaction and sella turcica bridging.
Arcos-Palomino, Ustrell-Torrent 2019 [23]	Case–control study	To assess whether there was relationship between the degree of calcification of sella turcica and the presence or absence of an alteration in the tooth eruption direction using panoramic and cephalometric radiographs.	30 subjects with canine or premolars impaction or transposition (cases) and 120 selected randomly with absent altered direction of dental eruption (controls).	10–50	Sella turcica bridging	Premolars and canines impaction or transposition	Duplicate tracings were made by the same author on 20 films on two separate occasions with 15-day interval between tracings to assess the random error.	Subjects with altered direction of canine eruption showed a higher occurrence of sella turcica bridging than controls (the occurrence of partial and complete bridging: cases 76.6%, controls 40.8%).
Alqahtani 2019 [24]	Case–control study	To compare sella turcica bridge among orthodontic patients with congenitally missing maxillary lateral incisors (CMMLI) using panoramic and cephalometric radiographs.	49 patients with (study group) and 49 without complete dentition (control group).	12–43	Sella turcica bridging	Congenitally missing maxillary lateral incisors (CMMLI)	No data.	Patients with CMMLI tend to have a significantly higher frequency of sella bridging (the occurrence of partial and complete bridging: study group 69.4%, control group 46.9%).
Divya et al. 2018 [25]	Case–control study	To check frequency of sella turcica bridging in participants with impacted canines and hyperdontia compared with a control group using panoramic and cephalometric radiographs.	62 orthodontic patients with impacted canines and hyperdontia (study group) and 36 orthodontic patients without the presence of any dental anomaly (control group).	Mean age: Impacted canine 16.92 Hyperdontia 18.87 Control 17.56	Sella turcica bridging	Impacted canines and hyperdontia	25 randomly selected radiographs were retraced and measured after 2 weeks.	The presence of partial and complete bridging is significantly increased in patients with dental anomalies versus control group (the occurrence of partial and complete bridging: patients with impacted canine 61,5%, with hyperdontia 43.4%, control group 25%).
Scribante et al. 2017 [26]	Case–control study	To find any association between canine impaction, hyperdontia or hypodontia and sella bridging using panoramic and cephalometric radiographs.	163 patients with dental abnormalities—study group (78 patients with impacted canines, 68 with dental agenesis and 17 with hyperdontia), 47 subjects without dental abnormalities (control group).	No data	Sella turcica bridging	Canine impaction, hyperdontia or hypodontia	The same operator re-traced 20 randomly selected radiographs after a period of 6 weeks.	The presence of partial and complete bridging is significantly increased in patients with dental abnormalities versus control group (vestibular impacted canines 73%, palatal displaced canines 69%, congenital absence of upper lateral incisors 66%, hyperdontia 59%, lower second premolars hypodontia 58%, control group 57%).
Ali et al. 2014 [27]	Case–control study	To test whether an association exists between sella bridging and impacted canines using panoramic and cephalometric radiographs.	31 patients with palatally impacted canines (study group) and 70 with erupted canines (control group).	14–30	Sella turcica bridging	Impacted canines	30 randomly selected lateral radiographs were retraced and reevaluated by the principal investigator 2 weeks after initial analysis.	The frequency of sella turcica bridging is increased in subjects with impacted canines (the occurrence of partial and complete bridging: study group 80.6%, control group 51.4%).
Leonardi et al. 2011 [28]	Case–control study	To determine association between tooth transposition and bridging of the sella turcica using panoramic and cephalometric radiographs.	21 subjects with maxillary or mandibular dental transposition (study group) and 70 without dental abnormalities.	Mean age: Study group 14.5, Control group 13.8	Sella turcica bridging	Maxillary or mandibular dental transposition	Duplicate tracings of 10 radiographs were made on two separate occasions by the same author with a 2-week interval between tracings.	Subjects with calcification in the region of sella are at potential risk of developing dental transposition (the occurrence of partial bridge: study group 42.9%, control group 68.6% and complete bridging: study group 23.8%, control group 5.7%).
Sato, Endo 2019 [29]	Case–control study	To investigate the association between bridging of sella turcica and tooth agenesis using panoramic and cephalometric radiographs.	96 patients with tooth agenesis (study group), 32 without dental abnormalities (control group).	Age groups (mean age): Group A (under 14 years) 10.3 Group B (beyond 14 years) 18.5	Sella turcica bridging	Agenesis of second premolars or five or more teeth (the agenesis group)	Second measurement was performed by the same investigator who randomly selected 40 cephalograms 1 month after the first examination.	Maxillary second premolar agenesis and severe tooth agenesis had a higher prevalence of sella turcica bridging relative to the controls. However, the severity of tooth agenesis does not correspond to the severity of sella turcica bridging.
Leonardi et al. 2006 [30]	Case–control study	To investigate whether congenital absence of the second mandibular premolar, or the presence of palatally displaced canine (PDC), is associated with sella bridging using panoramic and cephalometric radiographs.	34 subjects with dental anomalies (study group) and 101 without dental abnormalities (control group).	8–16	Sella turcica bridging	Congenital absence of the second mandibular premolar, or palatally displaced canine (PDC)	Duplicate tracings of 20 films were made on two separate occasions by the same author with 2-week interval between tracings.	The prevalence of sella turcica bridge in adolescents with dental anomalies is increased (the occurrence of partial and complete bridging: study group 76.5%, control group 43.6%).

**Table 2:** Evaluation of case–control studies according to Newcastle–Ottawa quality assessment.

**Study**		**Baidas et al. 2018** [21]	**Ortiz et al. 2018** [22]	**Arcos-Palomino, Ustrell-Torrent 2019** [23]	**Divya et al. 2018** [25]	**Scribante et al. 2017** [26]
**Selection**	Is the case definition adequate?	1	1	1	1	1
	Representativeness of the cases	Not described properly	1	1	0	1
	Selection of Controls	1	1	1	0	1
	Definition of Controls	Not described properly	1	1	1	1
**Comparability**	Comparability of cases and controls on the basis of the design or analysis	2	2	2	1	2
		The authors standardized the procedure of rx taking, the evaluation of landmark identification and examination of sella turcica in both groups. Proper intraexaminer reliability assessment as well as blinding of examiner was performed.	The authors standardized the procedure of rx taking, the evaluation of landmark identification and examination of sella turcica in both groups. Proper intraexaminer reliability assessment as well as blinding of examiner was performed.	The authors standardized the procedure of rx taking, the evaluation of landmark identification and examination of sella turcica in both groups. Proper intraexaminer reliability assessment as well as blinding of examiner was performed.	The authors standardized the evaluation of landmark identification and examination of sella turcica in both groups. However, they were described poorly. The measurements were not repeated. The authors pooled together dental abnormalities of different etiology. The study groups were too small to assure low risk of results distortion.	The authors standardized the procedure of rx taking, the evaluation of landmark identification and examination of sella turcica in both groups. Intraexaminer reliability was verified.
**Outcome**	Ascertainment of exposure	1	1	1	1	1
	Same method of ascertainment for cases and controls	1	1	1	1	1
	Non-response rate	1	1	1	1	1
**Total**		**7**	**9**	**9**	**6**	**9**
**Study**		**Ali et al. 2014** [27]	**Leonardi et al. 2011** [28]	**Sato, Endo 2019** [29]	**Leonardi et al. 2006** [30]	**Alqahtani 2019** [24]
**Selection**	Is the case definition adequate?	1	1	1	1	1
	Representativeness of the cases	0	0	1	1	1
	Selection of Controls	1	1	1	1	1
	Definition of Controls	1	1	1	1	1
**Comparability**	Comparability of cases and controls on the basis of the design or analysis	1	1	2	2	1
		The authors standardized the procedure of rx taking, the evaluation of landmark identification and examination of sella turcica in both groups. Intraexaminer reliability was verified. The study group is much smaller than control group.	The authors standardized the procedure of rx taking, the evaluation of landmark identification and examination of sella turcica in both groups. Intraexaminer reliability was verified. The study group is much smaller than control group.	The authors standardized the procedure of rx taking, the evaluation of landmark identification and examination of sella turcica in both groups. Intraexaminer reliability was verified.	The authors standardized the procedure of rx taking, the evaluation of landmark identification and examination of sella turcica in both groups. Intraexaminer reliability was verified.	The author standardized the evaluation of landmark identification and examination of sella turcica in both groups. However, no measures decreasing the possible risk of bias were applied in the design of the study.
**Outcome**	Ascertainment of exposure	1	1	1	1	1
	Same method of ascertainment for cases and controls	1	1	1	1	1
	Non-response rate	1	1	1	1	1
**Total**		**7**	**7**	**9**	**9**	**8**

**Table 3:** Percentage of dental abnormalities in study groups and significance of the difference from general population.

Abnormality/Values	Mean (%)	SD (%)	*t*-Value	*p*-Value
PDC	30.02	13.73	4.141	0.0054
Hypodontia	40.11	22.39	2.856	0.0498

**Table 4:** Prevalence of complete sella turcica bridge in healthy individuals (in chronological order from earliest to most recent).

Author, Year	Study Material	Prevalence
Leonardi et al. (2011) [28]	70 cephalograms of Caucasian subjects	5.70% (*n* = 4)
Axselsson et al. (2004) [34]	72 cephalograms of healthy Norwegian individuals	11.11% (*n* = 8)
Kucia et al. (2014) [35] Dixit et al. (2017) [36]	322 cephalograms of Polish orthodontic patients 473 cephalograms of Nepali subjects	4.97% (*n* = 16) 6.77% (*n* = 32)
Konwar et al. (2016) [38]	100 cephalograms	4.00% (*n* = 4)
Camp (1924) [39]	110 skulls of deceased people	4.50% (*n* = 5)
Carstens (1949) [40]	461 cephalograms	4.60% (*n* = 21)
Bush (1951) [41]	343 skulls of deceased people	1.46% (*n* = 5)
Cederberg et al. (2003) [42]	255 lateral cephalometric radiographs	8.2% (*n* = 21)
Jones (2005) [43]	150 cephalograms of English orthodontic patients	7.33% (*n* = 11)
Leonardi et al. (2006) [30]	101 healthy Caucasian individuals (without dental anomalies)	9.90% (*n* = 10)
Alkofide (2007) [44]	180 cephalograms of Saudi patients with all skeletal classes	1.10% (*n* = 2)
Dasgupta et al. (2018) [45]	205 cephalograms of Indian patients	1.46% (*n* = 3)
Shrestha et al. (2018) [46]	120 cephalograms of Nepali patients	11.67% (*n* = 14)

**Table 5:** Prevalence of complete sella turcica bridge in individuals with dental anomalies (in chronological order from earliest to most recent).

Author, Year	Study Material	Prevalence
Leonardi et al. 2006 [30]	34 cephalograms of Caucasian adolescents with dental anomalies	17.60% (*n* = 6)
Leonardi et al. 2011 [28]	21 cephalograms of Caucasian subjects with dental transposition	33.30% (*n* = 7)
Ali et al. 2014 [27]	31 Pakistani orthodontic patients with maxillary palatal canine impactions (cephalograms)	25.80% (*n* = 8)
Scribante et al. 2017 [26]	Lateral cephalograms from 78 patients with impacted canines, 68 with dental agnesis and 17 with hyperdontia	9.20% (*n* = 15)
Divya et al. 2018 [25]	39 patients with imapacted canines and 23 patients with hyperdontia (cephalograms)	19.35% (*n* = 12)
Ortiz et al. 2018 [22]	38 CBCT images of patients with palatal canine impaction	7.90% (*n* = 3)
Baidas et al. 2018 [21]	62 cephalometric radiographs of patients with palatally imapacted canine	6.45% (*n* = 4)
Alqahtani 2019 [24]	49 cephalograms of subjects with congenital missing maxillary lateral incisors (CMMLI)	8.16% (*n* = 4)

## Data Availability

The data is available upon request.

## Abbreviations

CMMLI	congenitally missing lateral incisors
CBCT	cone beam computed tomography
PDC	palatally displaced canines

## References

[1] Tekiner H., Acer N., Kelestimur F. (2015). Sella turcica: An anatomical, endocrinological, and historical perspective. Pituitary.

[2] Sathyanarayana H.P., Kailasam V., Chitharanjan A.B. (2013). Sella turcica-Its importance in orthodontics and craniofacial morphology. Dent. Res. J..

[3] Pisaneschi M., Kapoor G. (2005). Imaging the sella and parasellar region. Neuroimaging Clin. N. Am..

[4] Kjær I. (2015). Sella turcica morphology and the pituitary gland-a new contribution to craniofacial diagnostics based on histology and neuroradiology. Eur. J. Orthod..

[5] Russell B.G., Kjaer I. (1999). Postnatal structure of the sella turcica in Down syndrome. Am. J. Med. Genet..

[6] Sathyanarayana H.P., Kailasam V., Singh G. (2013). The Size and Morphology of Sella Turcica in Different Skeletal Patterns among South Indian Population: A Lateral Cephalometric Study. J. Indian Orthod. Soc..

[7] Miletich I., Sharpe P.T. (2004). Neural crest contribution to mammalian tooth formation. Birth Defects Res. Part C Embryo Today Rev..

[8] Belmehdi A., Chbicheb S. (2019). Oral disorders related to acromegaly. Pan Afr. Med. J..

[9] Al-Ani A.H., Antoun J.S., Thomson W.M., Merriman T.R., Farella M. (2017). Hypodontia: An Update on Its Etiology, Classification, and Clinical Management. Biomed. Res. Int..

[10] Gracco A.L.T., Zanatta S., Valvecchi F.F., Bignotti D., Perri A., Baciliero F. (2017). Prevalence of dental agenesis in a sample of Italian orthodontic patients: An epidemiological study. Prog. Orthod..

[11] Lakshmanan L., Gurunathan D. (2019). Prevalence of congenitally missing second premolar teeth in the Dravidian population. J. Forensic Dent. Sci..

[12] Badrov J., Lauc T., Nakaš E., Galić I. (2017). Dental Age and Tooth Development in Orthodontic Patients with Agenesis of Permanent Teeth. Biomed. Res. Int..

[13] Mallineni S.K., Nuvvula S., Cheung A., Kunduru R. (2014). A comprehensive review of the literature and data analysis on hypo-hyperdontia. J. Oral Sci..

[14] Yilmaz H.H., Türkkahraman H., Sayin M.O. (2005). Prevalence of tooth transpositions and associated dental anomalies in a Turkish population. Dentomaxillofac. Radiol..

[15] Meaney S., Anweigi L., Ziada H., Allen F. (2012). The impact of hypodontia: A qualitative study on the experiences of patients. Eur. J. Orthod..

[16] Rakhshan V. (2015). Congenitally missing teeth (hypodontia): A review of the literature concerning the etiology, prevalence, risk factors, patterns and treatment. Dent. Res. J..

[17] Park M.K., Shin M.K., Kim S.O., Lee H.S., Lee J.H., Jung H.S., Song J.S. (2017). Prevalence of delayed tooth development and its relation to tooth agenesis in Korean children. Arch. Oral. Biol..

[18] Moher D., Liberati A., Tetzlaff J., Altman D.G., PRISMA Group (2009). Preferred reporting items for systematic reviews and meta-analyses: The PRISMA statement. PLoS Med..

[19] Higgins J.P.T., Green S. (2011). Cochrane Handbook for Systematic Reviews of Interventions.

[20] Wells G., Shea B., O’Connell D., Peterson J.E., Welch V. (2011). The Newcastle-Ottawa Scale (NOS) for assessing the quality of case-control studies in meta-analyses. Eur. J. Epidemiol..

[21] Baidas L.F., Al-Kawari H.M., Al-Obaidan Z., Al-Marhoon A., Al-Shahrani S. (2018). Association of sella turcica bridging with palatal canine impaction in skeletal Class I and Class II. Clin. Cosmet. Investig. Dent..

[22] Ortiz P.M., Tabbaa S., Flores-Mir C., Al-Jewair T. (2018). A CBCT Investigation of the Association between Sella-Turcica Bridging and Maxillary Palatal Canine Impaction. Biomed. Res. Int..

[23] Arcos-Palomino I., Ustrell-Torrent J.M. (2019). Association between sella turcica bridging and altered direction of dental eruption: A case-control study. J. Clin. Exp. Dent..

[24] Alqahtani H. (2020). Association between sella turcica bridging and congenitally missing maxillary lateral incisors. J. Dent. Sci..

[25] Divya S., Urala A.S., Prasad G.L., Pentapati K.C. (2018). Sella turcica bridging a diagnostic marker for impacted canines and supernumerary teeth. J. Int. Oral. Health.

[26] Scribante A., Sfondrini M.F., Cassani M., Fraticelli D., Beccari S., Gandini P. (2017). Sella turcica bridging and dental anomalies: Is there an association?. Int. J. Paediatr. Dent..

[27] Ali B., Shaikh A., Fida M. (2014). Association between sella turcica bridging and palatal canine impaction. Am. J. Orthod. Dentofac. Orthop..

[28] Leonardi R., Farella M., Cobourne M.T. (2011). An association between sella turcica bridging and dental transposition. Eur. J. Orthod..

[29] Sato D., Endo T. (2020). Size and bridging of the sella turcica in Japanese orthodontic patients with tooth agenesis. Odontology.

[30] Leonardi R., Barbato E., Vichi M., Caltabiano M. (2006). A sella turcica bridge in subjects with dental anomalies. Eur. J. Orthod..

[31] Laganà G., Venza N., Borzabadi-Farahani A., Fabi F., Danesi C., Cozza P. (2017). Dental anomalies: Prevalence and associations between them in a large sample of non-orthodontic subjects, a cross-sectional study. BMC Oral Health.

[32] Dadgar S., Alimohamadi M., Rajabi N., Rakhshan V., Sobouti F. (2021). Associations among palatal impaction of canine, sella turcica bridging, and ponticulus posticus (atlas arcuate foramen). Surg. Radiol. Anat..

[33] Wak T.E., Akl R., Mati M., Khoury E., Ghoubril J. (2018). Association between sella turcica bridging and palatal canine impaction: Evaluation using lateral cephalograms and CBCT. Int. Orthod..

[34] Axelsson S., Storhaug K., Kjaer I. (2004). Post-natal size and morphology of the sella turcica. Longitudinal cephalometric standards for Norwegians between 6 and 21 years of age. Eur. J. Orthod..

[35] Kucia A., Jankowski T., Siewniak M., Janiszewska-Olszowska J., Grocholewicz K., Szych Z., Wilk G. (2014). Sella turcica anomalies on lateral cephalometric radiographs of Polish children. Dentomaxillofac. Radiol..

[36] Dixit S., Kafle D., Bornstein M., Sanjel S. (2017). Sella turcica bridging as a predicator of dentofacial anomalies: A cephalometric analysis. Orthod. J..

[37] Becktor J.P., Einersen S., Kjaer I. (2000). A sella turcica bridge in subjects with severe craniofacial deviations. Eur. J. Orthod..

[38] Konwar S.K., Singhla A., Bayan R. (2016). Morphological (Length, Depth, and Diameter) Study of Sella Turcica in Different Mandibular Growth Patterns in Indians. Int. J. Dent. Med. Spec..

[39] Camp J.D. (1924). The normal and pathological anatomy of the sella turcica as revealed by roentgenograms. Am. J. Roentgenol..

[40] Carstens M. (1949). Die Selladiagnostik. Fortschr. Geb. Rontgenstr. Nuklearmed..

[41] Busch W. (1951). Die morphologie der Sella turcica und ihre Beziehungen zur Hypophyse. Virchows Arch..

[42] Cederberg R.A., Benson B.W., Nunn M. (2003). Calcification of the interclinoid and petroclinoid ligaments of sella turcica: A radiographic study of the prevalence. Orthod. Craniofac. Res..

[43] Jones R.M., Faqir A., Millett D.T., Moos K.F., McHugh S. (2005). Bridging and dimensions of sella turcica in subjects treated by surgical-orthodontic means or orthodontics only. Angle Orthod..

[44] Alkofide E.A. (2007). The shape and size of the sella turcica in skeletal Class I, Class II, and Class III Saudi subjects. Eur. J. Orthod..

[45] Dasgupta P., Sen S., Srikanth H.S., Kamath G. (2018). Sella Turcica Bridging as A Predictor of Class II Malocclusion-An Investigative Study. J. Stomatol. Oral Maxillofac. Surg..

[46] Shrestha G.K., Pokharel P.R., Gyawali R., Bhattarai B., Giri J. (2018). The morphology and bridging of the sella turcica in adult orthodontic patients. BMC Oral Health.

[47] Motwani M.B., Biranjan R., Dhole A., Choudhary A.B., Mohite A. (2017). A study to evaluate the shape and size of sella turcica and its correlation with the type of malocclusion on lateral cephalometric radiographs. IOSR J. Dent. Med. Sci..

[48] Morassi M.L., Trimarchi M., Nicolai P., Gregorini G., Maroldi R., Specks U., Facchetti F. (2001). Cocaine, ANCA, and Wegener’s granulomatosis. Pathologica.

[49] Trimarchi M., Bellini C., Toma S., Bussi M. (2012). Back-and-forth endoscopic septoplasty: Analysis of the technique and outcomes. Int. Forum Allergy Rhinol..

[50] Capparè P., Tetè G., Sberna M.T., Panina-Bordignon P. (2020). The Emerging Role of Stem Cells in Regenerative Dentistry. Curr. Gene Ther..

[51] Crespi R., Capparé P., Romanos G.E., Mariani E., Benasciutti E., Gherlone E. (2011). Corticocancellous porcine bone in the healing of human extraction sockets: Combining histomorphometry with osteoblast gene expression profiles in vivo. Int. J. Oral Maxillofac. Implant..

[52] Zizzari V.L., Zara S., Tetè G., Vinci R., Gherlone E., Cataldi A. (2016). Biologic and clinical aspects of integration of different bone substitutes in oral surgery: A literature review. Oral Surg. Oral Med. Oral Pathol. Oral Radiol..

